# *Ralstonia solanacearum* Suppresses Tomato Root Growth by Downregulation of a Wall-Associated Receptor Kinase

**DOI:** 10.3390/plants12203600

**Published:** 2023-10-17

**Authors:** Sushuang Liu, Qi Xue, Shuying Zhu, Yanmin Liu, Huasong Zou

**Affiliations:** School of Life Sciences and Health, Huzhou College, Huzhou 313000, China; liu@zjhzu.edu.cn (S.L.); xueqi2@zjhzu.edu.cn (Q.X.); zhushuying@zjhzu.edu.cn (S.Z.); liuyanmin@zjhzu.edu.cn (Y.L.)

**Keywords:** *Ralstonia solanacearum*, *hrpB*, transcriptome analysis, *WAKL20*, root development

## Abstract

The root architecture of a range of host plants is altered in response to *Ralstonia solanacearum* infection. This work aimed to identify host genes involved in root development during *R. solanacearum* infection. A deficient mutant of the type III secretion system regulator *hrpB* was created in *R. solanacearum* GMI1000. The *hrpB* mutant was impaired in virulence but showed a similar suppressive effect as wild-type GMI1000 on tomato root development. Based on comparative transcriptome analysis, 209 genes were found that showed the same changed expression pattern in GMI1000 and *hrpB* mutant infected roots relative to uninoculated roots. Among them, the wall-associated receptor kinase *WAKL20* was substantially downregulated in GMI1000 and *hrpB* mutant infected roots. Knockdown of *WAKL20* led to a shorter primary root length and fewer lateral roots in tomato as well as in *Nicotiana benthamiana*. The *WAKL20* is a pivotal target suppressed by *R. solanacearum* to shape the altered root development during infection.

## 1. Introduction

*Ralstonia solanacearum* is a soil-borne bacterial pathogen that causes disastrous wilt disease in a strikingly broad range of hosts [1]. It moves toward the host’s root system by flagellar-driven motility, following sensing and chemotaxis toward root exudates [2,3]. Initially, the bacterium attaches to host surfaces in reversible and irreversible manners, which are dependent on polysaccharides, adhesion proteins, cell-surface appendages, and extracellular DNase [4]. *R. solanacearum* cells aggregate at the zone of root elongation and sites of emergence of lateral roots [5,6]. 

Once *R. solanacearum* has entered the root vascular tissue, vertical movement from roots to shoots, circular vascular bundle invasion, and radial apoplastic spread in the cortex are required for *R. solanacearum* to successfully invade hosts [7]. Colonization in the vasculature is spatially restricted and delayed in resistant tomato (*Solanum lycopersicum*) plants [8]. Wilt symptoms will appear if the *R. solanacearum* population exceeds 10^8^ CFU/g in the stems of susceptible tomato plants [9]. In xylem vessels of asymptomatic tolerant plant cultivars, *R. solanacearum* can live for extended periods at moderately high cell densities (10^4^ to 10^7^ CFU/g stem) [10,11]. *R. solanacearum* encounters stress conditions of oxygen and nutrient scarcity in xylem. Even though xylem sap has a low concentration of nutrients, its chemical component contains at least 118 known metabolites. Among them, 42 xylem metabolites can be used by *R. solanacearum* as a sole carbon or nitrogen source in vitro [12,13]. Glutamic acid serves as an active component required by *R. solanacearum* to promote colonization in the roots and stems of tomato plants [14].

Resistance to bacterial wilt is quantitatively controlled by polygenic loci in available tomato cultivars with resistance [15,16]. Only four out of 285 tomato accessions originating from 23 countries show high resistance against *R. solanacearum* [17]. In the resistant cultivar ‘Hawaii7996’, the loci *Bwr-6* (Chromosome 6) and *Bwr-12* (Chromosome 12) have been identified as two major quantitative trait loci contributing to resistance against bacterial wilt [18]. The single-nucleotide polymorphism marker *Solyc12g009690* is tightly linked to *Bwr-12*, which codes for a putative LRR receptor-like protein [19]. In comparative transcriptomics analyses of resistant and susceptible cultivars, defense-related genes generally showed genotype-specific regulation and expression differences after *R. solanacearum* infection [20,21]. 

The plant root system architecture is controlled by inherent genetic factors as well as biotic and abiotic factors. In response to *R. solanacearum* infection, an inhibition of primary root growth is usually found in different plant species. *R. solanacearum* GMI1000 and two *hrp* mutants (*hrpB* and *hrcR*) disturb the development of petunia root architecture by inhibiting lateral root elongation and provoking swelling of the root tips [22]. *R. solanacearum* GMI1000 and the *hrpB* mutant show suppression of root growth in *Arabidopsis thaliana*, while the *hrpG* mutant lost the ability to suppress root growth [23]. 

Although neither the *hrpB* nor *hrpG* mutant is able to induce the formation of root hairs in Arabidopsis, the *hrpB* mutant still inhibits root growth [22]. The transcriptional profiles of Arabidopsis roots during the early stages of interaction with *R. solanacearum* reveal sequential activation of multiple hormone-signaling cascades, including abscisic acid (ABA), auxin, jasmonic acid, and ethylene. Moreover, transcriptional changes of genes involved in primary root, lateral root, and root hair formation exhibit high temporal dynamics upon infection [24]. 

The purpose of this study was to identify the genes involved in the root architecture in response to *R. solanacearum* infection. Based on a comparative transcriptome analysis, we found that *SlWAKL20* was downregulated in tomato roots infected with wild-type *R. solanacearum* and the *hrpB* mutant. Knockdown of *WAKL20* led to severe effects on root development. These results advanced our understanding of the molecular events associated with the altered modulation of tomato root architecture by *R. solanacearum*. 

## 2. Results

### 2.1. Construction of a hrpB Insertion Mutant from Strain GMI1000 

**Figure 1:**
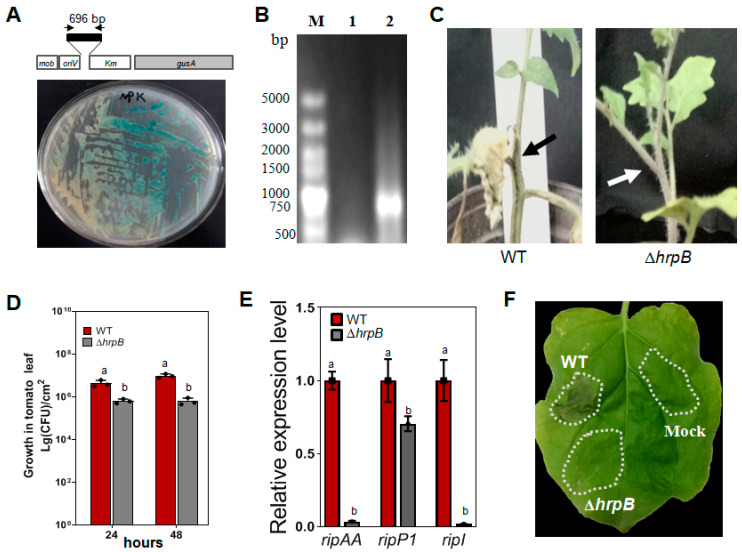
Phenotype analysis of the *hrpB* insertion mutant of GMI1000. (**A**) Creation of mutant Δ*hrpB* in GMI1000 by site-directed mutagenesis. The upper figure shows a 696 bp *hrpB* gene fragment cloned in the suicide plasmid pK19mobGII. The plates supplied with 20 μg/mL X-Gluc (lower panel) showed growth of Δ*hrpB* carrying an insertion of the suicide plasmid after genetic homologous recombination. (**B**) PCR identification of the GUS gene in mutant Δ*hrpB*. gusA-F and gusA-R primers were applied to amplify the 748 bp GUS gene from Δ*hrpB* carrying the integration of pK19mobGII in its genome. M, DL5000 DNA marker; 1, wild-type GMI1000; 2, Δ*hrpB*. (**C**) Virulence assay of mutant Δ*hrpB* on *Solanum lycopersicum*. A cell suspension of OD_600_ = 0.3 was dripped onto the freshly cut petiole surface of 4-week-old *S. lycopersicum* ‘Hongyangli’ plants. Wilt symptoms were scored at 4 dpi. The inoculated sites are indicated by arrows. (**D**) Growth in the tomato leaf. The bacterial cells were prepared to OD_600_ = 0.3 and infiltrated into *S. lycopersicum* ‘Hongyangli’ leaves. Leaf discs were collected from inoculated leaves at 24 and 48 h post infiltration, and the growth was counted as the number of cells per square centimeter. Values represent the means from three replicates. Means ± standard deviation are plotted. Different letters at each column indicate the significant difference (ANOVA, *p* = 0.05). (**E**) qRT-PCR analysis of *ripAA*, *ripP1*, and *ripI* in mutant Δ*hrpB*. Total RNA was extracted from the cells infiltrated into leaves of 4-week-old *S. lycopersicum* ‘Hongyangli’. The expression level in wild-type GMI1000 (WT) was set to 1, and the level in Δ*hrpB* was calculated relative to that. Values represent the means from three replicates. Means ± standard deviation are plotted. Different letters at each column indicate a significant difference (ANOVA, *p* = 0.05). (**F**) The loss of the hypersensitive reaction of Δ*hrpB* on *Nicotiana benthamiana*. The cultured wild-type GMI1000 genome and Δ*hrpB* cells were prepared to OD_600_ = 0.3 and infiltrated into *N. benthamiana* leaves. The photo was taken at 2 dpi. Infiltration of H_2_O was used as a mock treatment. The inoculation zones are indicted by dotted lines.

A 696 bp DNA fragment in the middle of the *hrpB* gene was inserted into the multiple cloning sites of pK19mobGII to create an insertion mutant (Figure 1A). After the construct was introduced into GMI1000, site-directed mutants were screened on NB plates + Km, and the GUS activity was examined on plates supplied with 20 μg/mL X-Gluc (Figure 1A). A set of primers specifically anchored to the GUS coding sequence was applied to verify the insertion of pK19mobGII in the mutant genome (Figure 1B). 

The insertion mutant Δ*hrpB* showed no pathogenicity on tomato plants, and its replication in tomato leaves was slower than wild-type GMI1000 (Figure 1C,D). After inoculation on tomato plants, *ripAA* and *ripI* were expressed in an *hrpB*-dependent manner, while *ripP1* was partially dependent on *hrpB* (Figure 1E). In addition, Δ*hrpB* completely lost the ability to induce a hypersensitive response in *N. benthamiana* (Figure 1F). 

### 2.2. The ΔhrpB Mutant Suppresses Tomato Primary Root Growth

**Figure 2:**
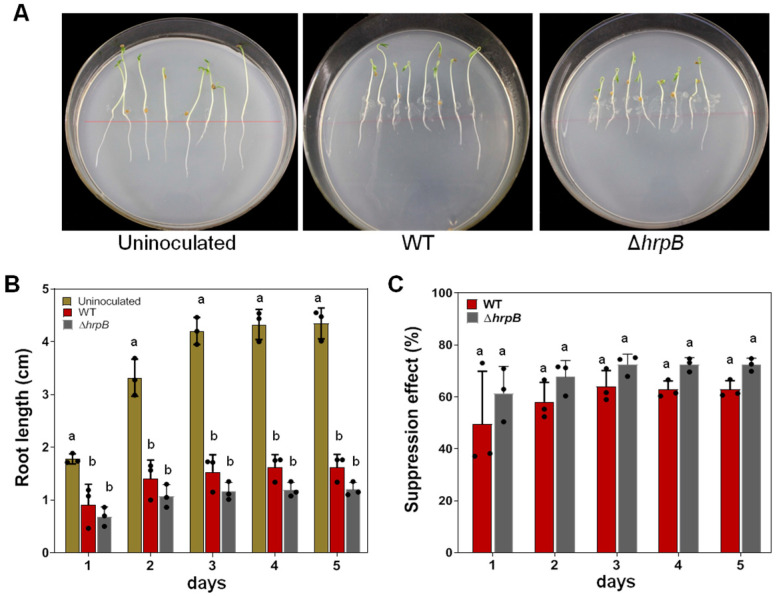
Δ*hrpB* exerted a suppression effect on tomato root growth. (**A**) Suppression of tomato root growth by Δ*hrpB*. Geminated seedlings of *Solanum lycopersicum* ‘Hongyangli’ were inoculated with a bacterial cell suspension (OD_600_ = 0.3) and then cultivated at 35 °C. The red line in every plate indicates the initial location of the root tip. The photos were taken at 3 dpi. (**B**) Primary root growth affected by Δ*hrpB*. The primary root length was detected at 1, 2, 3, 4, and 5 days post inoculation. Values represent the means from three replicates of 24 plants. Means ± standard deviation are plotted. Different letters at each column indicate a significant difference (ANOVA, *p* = 0.05). (**C**) Suppression effect on root growth by Δ*hrpB*. The data were calculated as reduced root length relative to uninoculated control plants. Values represent the means from three replicates of 24 plants. Means ± standard deviation are plotted. The letter at each column indicates no significant difference (ANOVA, *p* = 0.05).

To determine whether Δ*hrpB* affected tomato root growth, tomato cultivar ‘Hongyangli’ seedlings were inoculated with Δ*hrpB*. Compared with uninoculated control plants, Δ*hrpB* suppressed primary root growth (Figure 2A). At each time point of 1, 2, 3, 4, and 5 days post inoculation (dpi), the growing root length was 50% less than that of control plants (Figure 2B). Even though the root length of Δ*hrpB*-inoculated plants was somewhat shorter than that of GMI1000-inoculated plants, the difference was not statistically significant. The calculated suppression ratio of Δ*hrpB* showed no statistical difference from that of GMI1000 (Figure 2C). Therefore, the Δ*hrpB* retained the ability to suppress tomato primary root growth. 

### 2.3. Identification of Differentially Expressed Tomato Root Genes Affected by Both ΔhrpB and Wild-Type GMI1000

**Figure 3:**
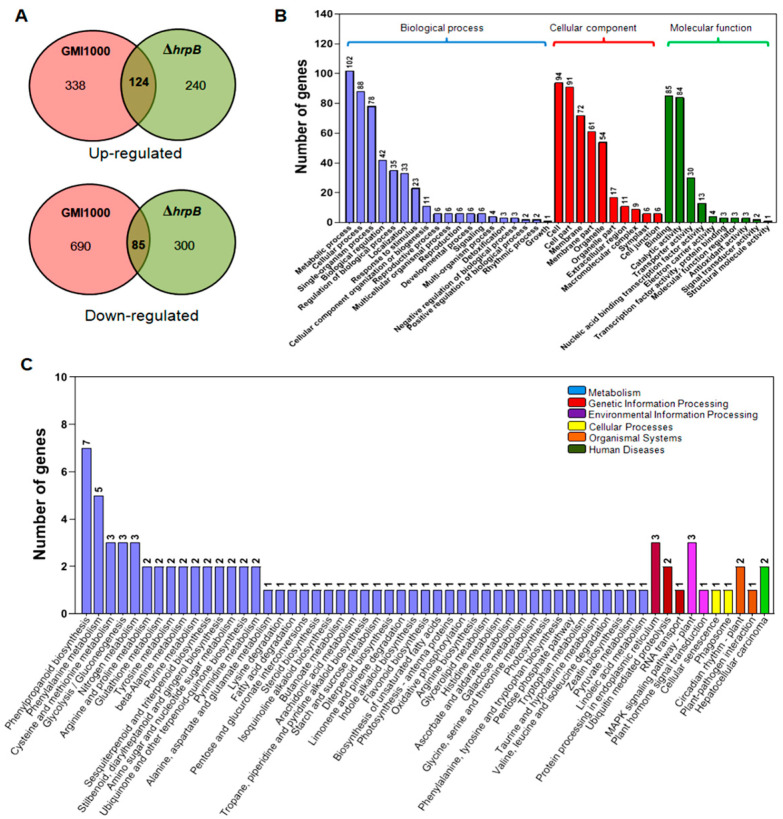
Transcriptome analysis of the differentially expressed genes in roots affected by GMI1000 and Δ*hrpB*. (**A**) Venn diagram of differentially expressed genes from pairwise comparisons between roots treated with wild-type GMI1000 and Δ*hrpB*. (**B**) Gene Ontology (GO) categories of the 209 differentially expressed genes found in roots treated with wild-type GMI1000 and Δ*hrpB*. The categories involved in Biological Process, Cellular Component, and Molecular Function are shown in blue, red, and green, respectively. (**C**) KEGG analysis of the 209 differentially expressed genes found in roots treated with wild-type GMI1000 and Δ*hrpB*. The categories involved in metabolism, genetic information processing, environmental information processing, cellular process, organismal systems, and human disease are shown in different colors.

As Δ*hrpB* and wild-type GMI1000 showed similar suppression effects on tomato primary root development, a comparative transcriptome analysis was employed to screen the genes potentially involved in root suppression. Compared with the uninoculated roots, 1028 differentially expressed genes (DEGs) were found in GMI1000-inoculated roots, including 338 upregulated and 690 downregulated genes (Table S1). Simultaneously, 540 DEGs were found from the roots inoculated with Δ*hrpB*, including 240 upregulated and 300 downregulated genes (Table S2). The close link analysis revealed that there were a total of 222 DEGs that were affected by both Δ*hrpB* and GMI1000. Among them, 13 genes showed diverse expression changes between Δ*hrpB*- and GMI1000-inoculated roots; therefore, these genes were omitted from the downstream analyses. The same change in expression was observed for 209 DEGs, with 124 upregulated and 85 downregulated genes (Table S3, Figure 3A). 

Among the 124 downregulated DEGs, 10 transporters were found to be involved in nitrate, potassium, ammonium, urea, proline, hexose, and nucleobase transportation, as well as a vacuolar amino acid transporter 1 (*Solyc09g098380*). Additionally, 1-aminocyclopropane-1-carboxylate synthase (*Solyc12g056180*), gibberellin 2-beta-dioxygenase 1 (*Solyc02g070430*), auxin efflux carrier component (*Solyc01g068410*), ABI3 protein isoform X1 (*Solyc06g083590*), and zeatin O-xylosyltransferase (*Solyc05g053400*) were closely related to plant hormone biosynthesis or signal transduction. Three Y nuclear transcription factors (*Solyc01g006930*, *NF-YA10*; *Solyc08g062210*, *NF-YA8*; *Solyc12g009050*, *NF-YA7*) and one subunit B (*Solyc01g067130*, *NF-YB5*) were downregulated in the roots inoculated with Δ*hrpB* or GMI1000. The downregulated DEGs included three serine/threonine-protein kinases (*Solyc03g078360*, *Solyc02g079540*, *Solyc12g094410*) and a wall-associated receptor kinase-like 20 (*SlWAKL20*, *Solyc01g100090*). The transcript level of *WAKL20* was extremely low in roots inoculated with GMI1000 and no expression was detected in the roots inoculated with Δ*hrpB* (Table S3). 

Among the 85 upregulated DEGs, *Solyc09g092470* and *Solyc01g101210* encode for the terpene synthase genes TPS14 and TPS35, respectively. The eight upregulated regulators consist of three MYB members (*Solyc07g026680*, *Solyc10g078720*, *Solyc06g005310*), nuclear transcription factor Y subunit B3 (*NY-B3*, *Solyc07g065500*), nuclear transcription factor Y subunit C (*NY-C*, *Solyc11g016920*), CAULIFLOWER A (*Solyc02g089210*), HHO2 (*Solyc05g009720*), and a two-component response regulator-like APRR1 (*Solyc03g115770*). For genes involved in the interaction with the host, two hypersensitive response-related genes *Solyc01g010770* and *Solyc03g033790*, transmembrane BAX inhibitor (*Solyc06g084130*), and wound-induced protein 1 (*Solyc06g050760*) were upregulated. Furthermore, pathogenesis-related PR4B (*Solyc01g097240*) and PR1 (*Solyc01g106630*) were found to be upregulated (Table S3). 

The 209 DEGs were mainly categorized into three terms in the Gene Ontology (GO) analysis: Biological Process, Cellular Component, and Molecular Function. In the Biological Processes term, there were 102 DEGs classified into metabolic processes and 88 DEGs classified into cellular processes. Most of the DEGs categorized into the Cellular Component term were classified into cell, membrane, and organelle. In the term Molecular Function, most of the DEGs were associated with binding and catalytic activity (Figure 3B). 

The 209 DEGs were broadly distributed in 57 pathways in the Kyoto Encyclopedia of Genes and Genomes (KEGG) analysis, including 47 pathways associated with Metabolism and three pathways associated with Genetic Information Processing. There were seven DEGs in the phenylpropanoid biosynthesis pathway, which was the category with the greatest number of DEGs in Metabolism. Five DEGs were identified in the phenylalanine metabolism pathway, which had the second-most DEGs in the KEGG analysis. Sixteen DEGs were distributed in the genetic information processing, environmental information processing, cellular processes, organismal systems, and human disease categories (Figure 3C). 

### 2.4. Downregulated WAKL20 Is Widely Distributed in Solanaceae Plants 

The transcriptome analysis identified four downregulated serine/threonine-protein kinase genes. Among them, *SlWAKL20* was transcribed at a low level in tomato roots inoculated with GMI1000, while it had no expression in the roots inoculated with Δ*hrpB*. 

**Figure 4:**
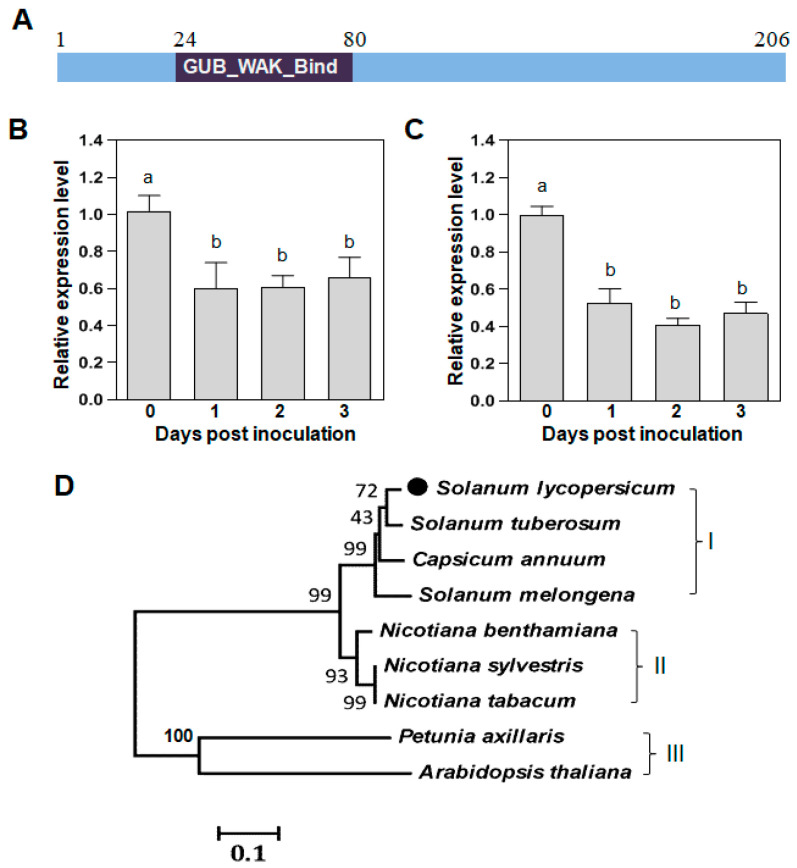
Sequence analysis of differentially expressed *SlWAKL20*. (**A**) Schematic diagram of the tomato SlWAKL20 structure. The conserved GUB_WAK_Bind domain is shown in black. (**B**) qRT-PCR analysis of the transcript level of *SlWAKL20* in tomato roots treated with wild-type GMI1000. Total RNA was extracted from the root samples at 1, 2, and 3 days post inoculation. The expression level in uninoculated roots was set to 1, and the levels in roots inoculated with GMI1000 were calculated relative to that. Values represent the means from three replicates. Means ± standard deviation are plotted. Different letters at each column indicate the significant difference (ANOVA, *p* = 0.05). (**C**) qRT-PCR analysis of transcript levels in tomato roots treated with mutant Δ*hrpB*. The results are the same as in (**B**). Values represent the means from three replicates. Means ± standard deviation are plotted. Different letters at each column indicate the significant difference (ANOVA, *p* = 0.05). (**D**) Phylogenetic tree using the Neighbor-Joining method constructed with amino acid sequences of WAKL20 homologs from nine representative *Ralstonia solanacearum* hosts. The confidence level was estimated using a bootstrap analysis. The evolutionary distances were computed using the Poisson correction method and are in the units of the number of amino acid substitutions per site. Evolutionary analyses were conducted in MEGA5.

In the tomato plant genome, WAKL20 is encoded by *Solyc01g100090* with a product of 206 amino acids. The cysteine-rich GUB_WAK_bind domain from 24 to 80 amino acids is an extracellular domain that binds to pectins in the cell wall (Figure 4A). 

qRT-PCR was conducted to evaluate the expression of *SlWAKL20* in response to GMI1000 and Δ*hrpB* infection. The results showed that the transcript level of *SlWAKL20* was significantly reduced in the roots inoculated with GMI1000 or Δ*hrpB* at 1, 2, and 3 days post inoculation (Figure 4B,C). For either strain inoculation, the suppression effect showed no difference among different time points.

SlWAKL20 homologs are highly conserved in Solanaceae species, including in *Solanum tuberosum*, *Capsicum annuum*, *S. melongena*, *N. tabacum*, and *N. benthamiana*. Their amino acids share 82–95% identity with tomato WAKL20. The homologs from *Petunia axillaris* and *Arabidopsis thaliana* share 41% and 38% identity with tomato SlWAKL20, respectively (Figure S1). A phylogenetic tree was constructed using WAKL20 homologs, and it revealed that WAKL20 from *S. lycopersicum*, *C. annuum*, *S. tuberosum*, and *S. melongena* were in one clade (Figure 4C). Three WAKL20 homologs from *Nicotiana* species were in a separate clade. WAKL20 from *P. axillaris* and *A. thaliana* were closely related and formed a clade distant from SlWAKL20 (Figure 4C). This suggested that WAKL20 was conserved in Solanaceae species but with distinctive diversity among *R. solanacearum* hosts. 

### 2.5. Knockdown of SlWAKL20 Resulted in Altered Root Development and Enhanced Resistance

**Figure 5:**
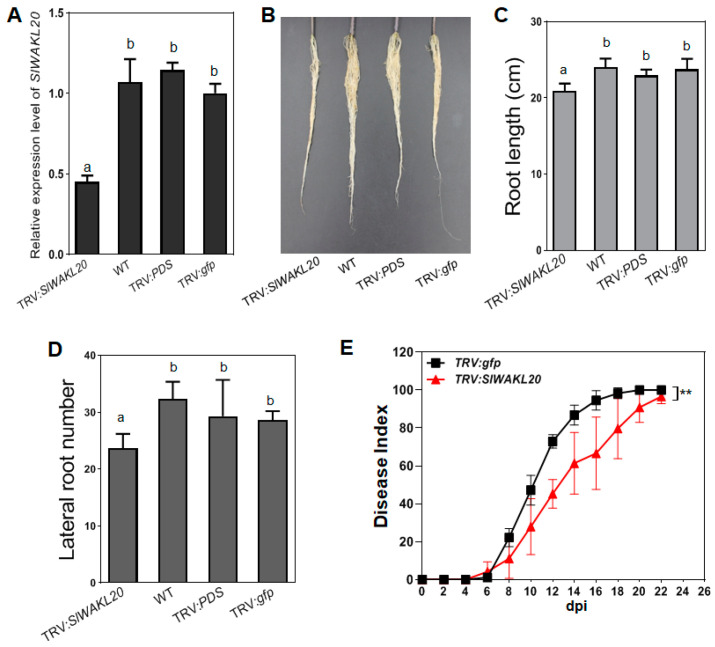
Silencing of the *SlWAKL20* gene in *Solanum lycopersicum*. (**A**) Detection of the transcript level of *SlWAKL20* in TRV-mediated silenced plants by qRT-PCR analysis. The transcript levels were evaluated when TRV:*PDS* plants showed a photobleaching phenotype. The transcript level in TRV:*gfp* plants was set to 1, and the levels in the silenced plants were calculated relative to that. Wild type and TRV:*PDS* plants were used as controls. Values represent the means from three replicates. Means ± standard deviation are plotted. Different letters at each column indicate the significant difference (ANOVA, *p* = 0.05). (**B**) Root growth of TRV:*SlWAKL20* plants. Root growth was scored when the control TRV:*PDS* plants showed a photobleaching phenotype. (**C**) Quantification of the reduced root length of TRV:*SlWAKL20* plants. Values represent the means from three repeats. Means ± standard deviation are plotted. Different letters at each column indicate the significant difference (ANOVA, *p* = 0.05). (**D**) Quantification of the number of lateral roots of TRV:*SlWAKL20* plants. Values represent the means from six replicates. Means ± standard deviation are plotted. Different letters at each column indicate the significant difference (ANOVA, *p* = 0.05). (**E**) Bacterial wilt disease development in SlWAKL20-silenced tomato plants. Disease symptoms were recorded every 2 days post inoculation with GMI1000. Each point represents the mean disease index of 24 plants (Student’s *t*-test, ** *p* < 0.01).

*SlWAKL20* was knocked down in tomato plants using TRV-mediated gene silencing. When the TRV:*PDS* control plants showed a photobleaching phenotype in their leaves, the expression of *SlWAKL20* was evaluated. Compared with the wild type, TRV:*PDS*, and TRV:*gfp* plants, the expression of *SlWAKL20* was reduced by 50% in TRV:*SlWAKL20* plants, demonstrating that *SlWAKL20* was successfully knocked down (Figure 5A). The knockdown of *SlWAKL20* resulted in a distinctive suppression of root growth (Figure 5B). Compared with the wild type and TRV:*gfp* plants, the root length of TRV:*SlWAKL20* plants was reduced by 3.1 cm, which was approximately 13% shorter than that of wild type plants (Figure 5C). In addition, TRV:*SlWAKL20* had an average of 23.7 lateral roots per plant, while the wild type plants had an average of 32.3 lateral roots per plant; the number of lateral roots was reduced by 26.7% (Figure 5D), indicating that knockdown of *SlWAKL20* led to an alteration of root architecture. 

To investigate the role of *SlWAKL20* in resistance against bacterial wilt, plants were inoculated with GMI1000 using the soil-soaking method. The *SlWAKL20*-silenced plants showed increased resistance to GMI1000 (Figure 5E).

### 2.6. Effect of Knockdown of NbWAKL20 on Root Development in N. benthamiana

**Figure 6:**
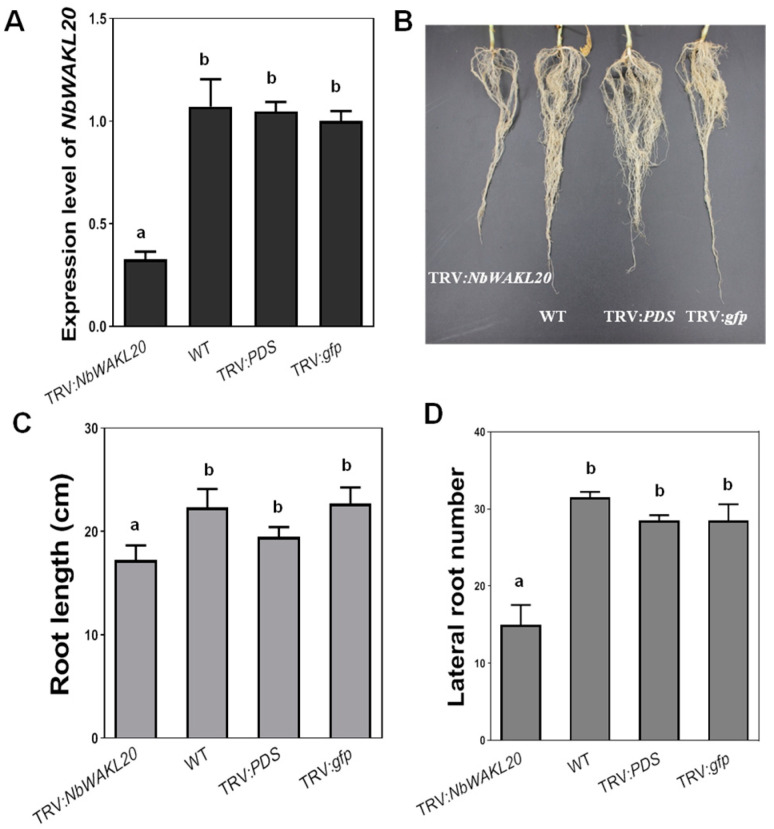
Silencing of NbWAKL20 in *Nicotiana benthamiana*. (**A**) Detection of the transcript level of *NbWAKL20* by qRT-PCR analysis. The transcript levels were evaluated when TRV:*PDS* plants showed a photobleaching phenotype. The transcript level in TRV:*gfp* plants was set to 1, and the levels in silenced plants were calculated relative to that. Wildtype and TRV:*PDS* plants were used as controls. Values represent the means from three replicates. Means ± standard deviation are plotted. Different letters at each column indicate a significant difference (ANOVA, *p* = 0.05). (**B**) Root growth of TRV:*NbWAKL20* plants. The root architecture was scored when the control TRV:*PDS* plants showed a photobleaching phenotype. (**C**) Quantification of the reduced root length of TRV:*NbWAKL20* plants. Values represent the means from three repeats. Means ± standard deviation are plotted. Different letters at each column indicate the significant difference (ANOVA, *p* = 0.05). (**D**) Quantification of the number of lateral roots of TRV:*NbWAKL20* plants. Values represent the means from six replicates. Means ± standard deviation are plotted. Different letters at each column indicate the significant difference (ANOVA, *p* = 0.05).

To evaluate the role of NbWAKL20 in root development, TRV-mediated gene silencing was conducted on *N. benthamiana*. The silencing efficiency was verified by qRT-PCR analysis of the transcript level of NbWAKL20 in TRV:*NbWAKL20* plants relative to the wild type, TRV:PDS, and TRV:gfp plants (Figure 6A). The root lengths of TRV:*NbWAKL20* plants were significantly reduced by 5.1 cm, which was approximately 16.2% shorter than that of wild type plants (Figure 6B). The number of lateral roots was reduced by approximately 50% relative to the control plants (Figure 6C). The knockdown of *NbWAKL20* resulted in similar effects on root length and lateral root number as in tomato. 

## 3. Discussion

Soil-born *R. solanacearum* attacks host plants by invading root systems, leading to foliar wilt symptoms, followed by rapid replication within the root xylem [1]. It has been reported that the root systems of *Medicago*, Arabidopsis, and *Petunia* show a particular response to *R. solanacearum* infection, including shortened roots and cell death at the root tips [22,23,25]. Based on the examination of root growth suppression by wild-type GMI1000 and Δ*hrpB*, we found that *R. solanacearum* showed type III-independent suppression of tomato primary root growth. Comparative transcriptome analysis revealed 209 DEGs with the same change in expression in tomato roots infected by GMI1000 and Δ*hrpB*. Downregulated *SlWAKL20* was identified as a target gene modulated by *R. solanacearum* to remodel the tomato root architecture. These results provided a deeper understanding of the root gene expression profile directing architecture remodeling upon *R. solanacearum* infection.

*hrpB* is an AraC-type transcriptional activator required for the induction of the *hrp* genes encoding the Hrp apparatus and most of the effector genes in plants [26,27]. Disruption of *hrpB* results in complete loss of virulence, as well as causing the inactivation of effector genes [28]. Even though the primary root growth of the host is usually arrested by *R. solanacearum*, variation was observed in response to mutant Δ*hrpB*. Δ*hrpB* fails to cause root growth arrest, root tip swelling, browning, and epidermis loss of viability in *Medicago truncatula* roots [25]. However, similar to the wild type, Δ*hrpB* disturbs the development of root architecture in petunia by inhibiting lateral root elongation and provoking the swelling of the root tips [22]. To evaluate the role of *hrpB* in tomato root growth arrest, an insertion mutant of Δ*hrpB* was created in this study. Phenotypic alterations of tomato and tobacco plants, as well as the induction of three representative effector genes (*ripAA*, *ripP1*, and *ripI*) were consistent with previous research [27,29]. Most importantly, the mutant retained the ability to suppress primary root growth, similar to the previously described interaction with petunia roots [22]. 

A number of DEGs have been identified from roots of Arabidopsis, tomato, and the wild potato *Solanum comersonii* upon *R. solanacearum* infection, including those related to transcription regulation, hormone biosynthesis, transport, and signal transduction [21,24,30]. The most attractive DEGs are those involved in hormone signal transduction. Deep sequencing on early reprogrammed Arabidopsis root revealed a sequential activation of multiple hormone signaling pathways, such as ABA, auxin, jasmonic acid, and ethylene [24]. The auxin and ethylene signaling genes were induced by *R. solanacearum* infection. ABA and jasmonic acid-associated genes are induced in the early infection stage and down-regulated in the late infection stage. The interruption of ABA signaling promotes leaf wilting symptom development but shows no effect on root structural changes [24]. 

As the Δ*hrpB* shows similar suppressive effects on tomato primary root development as wild type GMI1000, we conducted a comparative transcriptome analysis to screen the DEGs with the same expression changes in GMI1000- and Δ*hrpB*-infected roots. Compared with uninoculated roots, 209 genes showed the same expression change in GMI1000- and Δ*hrpB*-infected roots. Five hormone biosynthesis and signal transduction associated genes, 1-aminocyclopropane-1-carboxylate synthase (*Solyc12g056180*), gibberellin 2-beta-dioxygenase 1 (*Solyc02g070430*), auxin efflux carrier component (*Solyc01g068410*), ABI3 protein isoform X1 (*Solyc06g083590*), and zeatin O-xylosyltransferase (*Solyc05g053400*), were downregulated, suggesting that ethylene, auxin, gibberellins, ABA, and zeatin signaling pathways were disturbed. The finding of the zeatin O-xylosyltransferase gene suggested a more complex hormone signaling pathway in tomato roots in response to *R. solanacearum* infection. 

Plant terpene synthases (TPS) are responsible for the biosynthesis of terpene skeletons to produce terpenoid natural products. The TPS gene family has been characterized from various plants, with approximately 20 to 150 genes in sequenced plant genomes [31]. There are 34 full-length TPS genes and 18 TPS pseudogenes in the tomato genome [32]. Biochemical analysis has identified the catalytic activities of all enzymes encoded by the 34 TPS genes: 1 isoprene synthase, 10 exclusively or predominantly monoterpene synthases, 17 sesquiterpene synthases, and 6 diterpene synthases [32]. TPS14 (*Solyc09g092470*) and TPS35 (*Solyc01g101210*) were upregulated in roots inoculated with GMI1000 or Δ*hrpB*. TPS14 catalyzes the biosynthesis of bisabolene, and TPS35 catalyzes the biosynthesis of guaia-1(10),11-diene and (Z,Z)-farnesol [32]. Plant terpenes represent a vital group of chemicals showing antimicrobial activities against bacteria, fungi, and viruses [33]. Flowers of *A. thaliana* lacking volatile (E)-β-caryophyllene emission show greater bacterial growth on their stigmas than wild-type flowers [34]. Even though the exact roles of their bio-products have not been experimentally defined in tomato, the upregulation of *TPS14* and *TPS35* suggested that secondary metabolites are affected by *R. solanacearum*. Secondary metabolites are not restricted to terpene biosynthesis. Compared with susceptible cultivars, a total of 41 metabolites are prominent in the two highly resistant tomato cultivars, including amino acids, organic acids, lipids, derivatives of cinnamic acid and benzoic acids, flavonoids, and steroidal glycoalkaloids [35].

*R. solanacearum* infection induces the expression of a set of transcription regulators, including two downregulated WRKY members (*WRKY40b*, *WRKY51*) and three upregulated MYB members (*MYBS3*, *MYBPHL5*, *MYB48*). In pepper plants, *WRKY40b* is a negative regulator in response to *R. solanacearum* infection by modulating defense genes [36]. Nuclear transcription factor Y (NF) is a trimeric regulator complex consisting of three subunits: NF-YA, NF-YB, and NF-YC [37]. NF-YB and NF-YC form a heterodimer via interacting histone fold domains, which generates a surface for the NF-YA accession to generate a NF-YA/NF-YB/NF-YC trimeric complex [38]. The binding ability of the CCAAT-motif of NF-YA allows the complex regulator to regulate downstream targets depending on the transcriptional activation activities of NF-YB and NF-YC [38]. In *Manihot esculenta*, the NF-Y regulator complex positively regulates the expression of pathogenesis-related genes, contributing to the plant defense response to the bacterial pathogen *Xanthomonas axonopodis* pv. *manihotis* [39]. A total of 59 NF-Y genes, including 10 NF-YA, 29 NF-YB, and 20 NF-YC genes, are found in the tomato genome [40]. In response to GMI1000 or Δ*hrpB* infection, NF-YB3 (*Solyc07g065500*) and NF-YC4 (*Solyc11g016920*) were upregulated. Three NF-YA genes (*Solyc01g006930*, *NF-YA10*; *Solyc08g062210*, *NF-YA8*; *Solyc12g009050*, *NF-YA7*) and *NF-YB5* (*Solyc01g067130*) were downregulated. *NF-YB3* (*Solyc07g065500*) and *NF-YA8* (*Solyc08g062210*) have been reported to play roles in fruit ripening [40]. This suggests that the NF-Y regulator exerts multiple regulatory roles in tomato plants.

Plant cell wall-associated kinases (WAKs) and wall-associated kinase-like (WAKL) are receptor-like kinases that are widely present in plant species [41]. These kinases possess wall-associated receptor kinase galacturonan-binding (GUB-WAK-bind) and epidermal growth factor domains appearing more often in WAK proteins, enabling them to sense the extracellular environment [42]. *Brassica napus* possesses the WAKL protein Rlm9, which confers a race-specific resistance against *Leptosphaeria maculans* carrying the corresponding avirulence gene *AvrLm5-9* [43]. The wheat *Stb6* gene encodes a WAKL protein specifying a gene-for-gene disease resistance to foliar fungal pathogen *Zymoseptoria tritici* [44]. The tomato genome has at least 11 *SlWAK* and 18 *SlWAKL* genes in an uneven distribution in 9 of the 12 chromosomes [45]. The tomato gene *Solyc01g100090* coding for *SlWAKL20* was substantially suppressed in the roots inoculated with GMI1000 or Δ*hrpB*. Knockdown of *SlWAKL20* resulted in shortened primary root development and fewer lateral roots, suggesting a critical role in root development. Moreover, knockdown of *SlWAKL20* resulted in enhanced resistance to *R. solanacearum*. Homologs of *SlWAKL20* in other Solanaceae species may play similar roles, as knockdown of *NbWAKL20* led to the same phenotypic alterations in *N. benthamiana*. Hence, WAKL20 is a pivotal target gene affected by *R. solanacearum* to remodel the tomato root architecture.

In general, 209 differentially expressed genes were identified based on comparative transcriptome analysis of tomato roots in response to *R. solanacearum* infection. Among them, *WAKL20* is a pivotal target suppressed by *R. solanacearum* to shape the alteration of root development during infection.

## 4. Materials and Methods

### 4.1. Bacterial Strains and Plasmids

The bacterial strains and plasmids used in this study are listed in Table S4. *R. solanacearum* was cultivated in nutrient broth (NB) medium or NB plate with 1.5% agar (NA) at 28 °C [46]. *Escherichia coli* was cultured in Luria-Bertani medium (LB) at 37 °C. Antibiotics were applied at the following concentrations: kanamycin (Km), 50 μg/mL; polymyxin B (PB), 50 µg/mL.

### 4.2. Plants and Cultivation

*N. benthamiana* and *S. lycopersicum* L. cv. Hongyangli seeds were germinated and cultivated in a greenhouse under a 16 h light/8 h dark photoperiod at 25 °C.

### 4.3. Construction of an hrpB Insertion Mutant

A 696 bp DNA fragment of the *hrpB* gene was PCR amplified using the primers hrpB-F and hrpB-R (Table S5). The PCR product was cloned into the suicide vector pK19mobGII at the *Eco* RI and *Pst* I cloning sites, generating pK19-hrpB (Table S4). The vector pk19mobGII harbors a constitutively expressed *GUS* gene, allowing for the determination of plasmid integration by simply observing the color of the colonies [47]. After transformation into *Escherichia coli* S17-1, pK19-hrpB was introduced into wild-type GMI1000 by biparental mating. The mutants with pK19-hrpB integrated into their genome were selected on NA plates containing the antibiotics Km and PB. GUS activity in the obtained Δ*hrpB* mutants was then examined on NA plates supplied with 20 μg/mL 5-bromo-4-chloro-3-indoxyl-b-D-glucuronide (X-gluc). To further verify plasmid integration, the primers gusA-F and gusA-R were used to amplify a 748 bp product of the GUS gene from the mutant genomic DNA (Table S5).

### 4.4. Bacterial Virulence, Replication, and Hypersensitive Response Induction Assays

The wild-type GMI1000 and Δ*hrpB* were cultured in NB broth medium and then suspended in sterile distilled water to a final concentration of 10^8^ CFU/mL (OD600 = 0.3). Bacterial suspensions were inoculated on a stem wound created with a needle on four-week-old *S. lycopersicum* ‘Hongyangli’ plants. Wilt symptoms were scored and photographed at 4 dpi. For the replication assay *in planta*, the suspension was infiltrated into four-week-old *S. lycopersicum* ‘Hongyangli’ plant leaves. At 2 dpi, leaf discs were collected to count the cell number. Bacterial replication was represented as the cell formation unit (CFU) per square centimeter, and the standard deviation was calculated using colony counts from the three triplicate samples. Experiments were repeated three times.

### 4.5. Suppression of Tomato Root Growth by R. solanacearum

Seeds were sterilized with a solution containing 30% bleach and 0.02% Triton-X 100 for 10 min, washed three times with sterile distilled water, and germinated on 1.0% agar MS plates. After germination, the seedlings were transferred to new MS plates (eight seedlings per plant) and inoculated with 5 μL bacterial suspension (OD600 = 0.3) at every root tip. A line was made to indicate the initial site of the root tip on each plate. Then, the plates were sealed with Parafilm™ and placed in a greenhouse under constant conditions of 16 h light/8 h dark photoperiod at 25 °C. At 1, 2, 3, 4, and 5 dpi, the root length was measured with a ruler. The suppression ratio was calculated as the reduced length of the roots inoculated with *R. solanacearum* divided by the root length of the uninoculated control.

### 4.6. RNA-Seq Analysis

Whole roots from 10 plants inoculated with wild-type GMI1000 or Δ*hrpB* were harvested at 2 dpi. Plant roots treated with water were collected and used as the control. For every treatment, the samples were collected twice and pooled as a replicate, and three independent replicates were performed. The samples were submitted to Majorbio (Shanghai, China) for RNA-seq. After the samples were ground into a powder under liquid nitrogen for total RNA extraction using TRIzol (Invitrogen, Shanghai, China), 2 µg from each pool was subjected to RNA-seq on the Illumina Novaseq 6000. A total of 305,991,445 reads were generated after quality filtering and mapping. Reads for each of the samples were aligned to the ITAG2.4 *S. lycopersicum* reference genome. A differential gene expression analysis was performed using the DEseq2 package with an adjusted *p* value of 0.05 and a 2-fold change. A GO analysis was performed using Blast2GO (http://www.blast2go.com/) to assign the functional category on 20 May 2020. The GO categories shown are for Biological Process, Cellular Component, and Molecular Function. The metabolic pathways of the DEGs were analyzed according to the KEGG database (http://www.genome.jp/kegg/ accessed on 20 May 2020).

### 4.7. Real-Time Quantitative RT-PCR

Total RNAs were extracted using TRIzol (Invitrogen, Shanghai, China) according to the manufacturer’s protocol. The quality of the total RNA was analyzed by gel electrophoresis and the quantity was measured by a spectrophotometer. PCR was conducted on a CFX connect system (Bio-Rad, Hercules, California, CA, USA) with ChamQ SYBR qPCR Master Mix (Genstar, Beijing, China). The primers used for *ripAA*, *ripP1*, *rip1*, and *SlWAKL20* are listed in Table S5. The expression of *gyrA* was used as an internal reference to normalize the expression levels of three effector genes in *R. solanacearum*. The expression of *SlActin* and *NbEF1α* were used as the internal references to normalize the expression level of *WAKL20* in tomato and *N. benthamiana* plants, respectively (Table S5). The PCR thermal cycle conditions were as follows: denaturation at 95 °C for 30 s; 40 cycles of 95 °C for 5 s; 58 °C for 30 s. Relative expression levels and statistical analyses were conducted using CFX Maestro^TM^ software [48]. Each experiment was repeated three times.

### 4.8. Virus-Induced Gene Silencing

Virus-induced gene silencing was performed using TRV:*PDS* as the control to evaluate the silencing efficiency. A 300 bp partial sequence of *SlWAKL20* was cloned in the pTRV2 vector at *Eco* RI/*Bam* HI sites to generate TRV:*SlWAKL20*. TRV:*gfp* was used as a negative control [46]. A mixture of GV3101 cultures (1:1, *v*/*v*) that contained pTRV1 and pTRV2 constructs was co-infiltrated into the leaves of two-week-old *S. lycopersicum* ‘Hongyangli’. When the PDS-silenced plants exhibited a photo-bleached phenotype, qRT-PCR was performed to detect the transcripts of *SlWAKL20* to evaluate the knockdown efficiency. The root growth was then examined by measuring the whole root length and the number of lateral roots. The silencing experiments were repeated three times, with six independent plants for each repeat. The silencing of *NbWAKL20* was performed on *N. benthamiana* by using the same strategy.

Bacterial disease development on *SlWAKL20*-silenced and TRV:*gfp* plants was assessed by the soil-soaking inoculation method. The plants were drenched with a GMI1000 cell suspension to a final density of 5 × 10^7^ CFU/g of soil. After inoculation, the plants were placed at 28 °C and the disease progress was recorded on a 0 to 4 disease severity scale as follows: 0, no wilting; 1, 1 to 25% of leaves wilted; 2, 26 to 50% of leaves wilted; 3, 51 to 75% of leaves wilted; and 4, 76 to 100% of leaves wilted. All results are reported as the means from three assays, each containing eight plants for each treatment.

### 4.9. Sequence Analysis

The conserved domain in SlWAKL20 was predicted in NCBI (https://blast.ncbi.nlm.nih.gov/ accessed on 20 May 2020) using the Conserved Domain Search Service. Homolog sequences were retrieved from the NCBI database through a BLAST search. A Neighbor Joining phylogenetic tree was generated from the amino acid sequences by using the Poisson correction method with MEGA 5.0 [49]. A bootstrap consensus tree was inferred from 1000 replicates and was taken to represent the evolutionary history of the taxa analyzed. The consensus amino acid positions were generated using BioEdit (Borland Software Corporation, California, CA, USA).

### 4.10. Statistical Analysis

Statistical analyses were conducted with GraphPad Prism software (GraphPad Software, San Diego, CA, USA) with an unpaired one-way or two-way analysis of variance (ANOVA) at the 95% level with Tukey’s multiple comparison tests. The values are presented as means ± standard deviation.

## Data Availability

The RNA-seq dada are available in NCBI using the accession numbers SAMN31613169-SAMN31613186. All other raw data are available upon request to H.Z.

## Supplementary Materials

The following supporting information can be downloaded at: https://www.mdpi.com/article/10.3390/plants12203600/s1, Figure S1: Alignment of amino acid sequences of WAKL20 from *S. lycopersicum*, *N. benthamiana*, *N. sylvestris*, *N. tabacum*, *Capsicum annuum*, *S. melongena*, *S. tuberosum*, *P. axillaris*, and *A. thaliana*; Table S1: Differentially expressed genes in tomato roots inoculated with GMI1000; Table S2: Differentially expressed genes in tomato roots inoculated with Δ*hrpB*; Table S3: Differentially expressed genes in tomato roots affected by both GMI1000 and Δ*hrpB*; Table S4: Strains and plasmids used in this study; Table S5: Primers used in this study.
