# Supplementary material for: Ralstonia solanacearum Suppresses Tomato Root Growth by Downregulation of a Wall-Associated Receptor Kinase
Source: Plants (Basel). 2023 Oct 17;12(20):3600. doi: 10.3390/plants12203600 (PMC10610323; doi:10.3390/plants12203600)
Supplement: Supplementary file 1 [file plants-12-03600-s001.zip › Table S4 strains and plasmids used in this study.pdf]

Table S4. Bacterial strains and plasmids used in this study

Strains or plasmids	Relevant characteristics	Resources
**Strains**		
Ralstonia solanacearum		
GMI1000	Wild-type, phylotype I, biovar 3, race 1	[1]
Δ*hrpB*	Km^r, a insertion mutant of *hrpB* gene derived from GMI1000	This study
Escherichia coli		
DH5α	*F⁻ recA hsdR17 (rk⁻, mk⁺) ϕ80lacZΔM15*	Clontech
S17-1	λ*pir hsdR pro thi*;Tc::Mu Km::Tn*7*	Lab store
Agrobacterium tumefaciens		
GV3101	Rif^r, with Ti plasmid pMP90	Lab store
**Plasmids**		
pK19mobGII	Km^r, a suicide vector carrying a *gusA* reporter gene	[2]
pK19-hrpB	Km^r, a 696-bp *hrpB* gene fragment cloned in pK19mobGII	This study
TRV1	Km^r, a VIGS vector encoding the replication, movement and cysteine-rich protein protoeins of tobacco rattle virus	[3]
TRV2	Km^r, a VIGS vector harboring the coat protein and two non-structural proteins of tobacco rattle virus	[3]
TRV:*PDS*	Km^r, TRV2 carrying tomato *PDS*	[3]
TRV:*gfp*	Km^r, a 358-bp fragment of *gfp* gene cloned in TRV2	[4]
TRV: *SlWAKL20*	Km^r, a 300-bp *SlWAKL20* gene cloned in TRV2 at *Eco* RI/*Bam* HI sites	This study
TRV:*NbWAKL20*	Km^r, a 305-bp fragment of *Niben101Scf00369g24023.1* gene cloned in TRV2	This study

References

[1] Boucher, C.A.; Barberis, P.A.; Trigalet, A.P.; Demery, D.A. Transposon mutagenesis of *Pseudomonas solanacearum*: isolation of Tn*5*-induced avirulent mutants. *J. Gen. Microbiol.* **1985,**

131, 2449–2457.

[2] Katzen, F.; Becker, A.; Ielmini, M.; Oddo, C.G.; Ielpiet, L. New mobilizable vectors suitable for gene replacement in gram-negative bacteria and their use in mapping of the 3' end of the *Xanthomonas campestris* pv. *campestris* gum operon. *Appl. Environ. Microbiol.* **1999,** *65,* 278-282

[3] Liu, Y.; Schiff, M.; Dinesh-Kumar, S.P. Virus-induced gene silencing in tomato. *Plant J.* **2002,** *31,* 777-786.

[4] Sun, T.; Wu, W.; Wu, H.; Rou, W.; Zhou, Y.; Zhuo, T.; Fan, X.; Hu, X.; Zou, H.S. *Ralstonia solanacearum* elicitor RipX induces defense reaction by suppressing the mitochondrial *atpA* Gene in host plant. *Int. J. Mol. Sci.* **2020,** *21,* 2000.
