# Supplementary material for: Ralstonia solanacearum Suppresses Tomato Root Growth by Downregulation of a Wall-Associated Receptor Kinase
Source: Plants (Basel). 2023 Oct 17;12(20):3600. doi: 10.3390/plants12203600 (PMC10610323; doi:10.3390/plants12203600)
Supplement: Supplementary file 1 [file plants-12-03600-s001.zip › Table S5 Primers used for molecular cloning and qRT-PCR in this study.pdf]

Table S5. Primers used for molecular cloning and qRT-PCR in this study

Primer pair	Sequence(5'-3')	Cutting sites	Description or purpose
Primers used for molecular cloning			
hrpB-F/hrpB-R	TACGAATTCGCCGTGCAGGCCGGCA	*Eco* RI-*Pst*I	A 696-bp *hrpB* gene cloned in pK19mpbGII
	CCTGCAGCGCGCGCTCGGTCACGTT		
TRV-SlWAKL20-F/	GCGAATTCCCTTAGAGTTCTTGTCATCTGTGGG	*Eco* RI-*Bam* HI	A 300-bp fragment of *SlWAKL20* cloned in TRV2
TRV-SlWAKL20-F	GCGGATCCACAATGTGTTCTTACAGTTCCTTCC		
TRV-NbWAKL20-F/	CGGAATTCTGATTTATCTTTAGGTGGC/	*Eco* RI-*Bam* HI	A 305-bp fragment of *NbWAKL20* cloned in TRV2
TRV-NbWAKL20-R	TCGGATCCATGCACTAATGGAGTCTTCAG		

Primers used for PCR analysis

Gene	Sequence(5'-3')	Description
gusA-F/gusA-R	TCCTGTAGAAACCCCAACCC; CAGTTCATAGAGATAACCTTCACCC	748-bp of partial *GUS* gene
qRT-ripAA	GACAAGCGGCTGGGAATACA; GTGCTGGTCGGGATAAACAT	91-bp
qRT-ripP1	CTGGAGATGTCGGCGGTG; GCGCTTGGCGTCTTCATAG	138-bp
qRT-ripI	CAGTTGCGCCTCCATGAGT; GCTCGGTCTTCAGGTTCCA	91-bp
qRT-gyrA	CGACTGGAACCGTCCCTAC; TCCGCACGATGGTGTCATA	104-bp
qRT-SlWAKL20	AACATATCTTCGCGTAATACGGTCA;CTTCATTAGCTTCCTCGAATCGCCT	92-bp
qRT-SlActin	ATGTTGCTATTCAGGCGGTTTTGTC;CAGGATTTTCATCAGACAGTCAGTA	182-bp
qRT-NbEF1α	TGGTGTCCTCAAGCCTGGTATGGTTG;ACGCTTGAGATCCTTAACCGCAACATTCTT	160-bp
qRT-NbWAKL20	CTTAGAGTTCTTGTCCTCCGTAGGA;GTATGGAAGGAGGGCTAATAACAAG	92-bp
