# Supplementary figures and images for: Ralstonia solanacearum Suppresses Tomato Root Growth by Downregulation of a Wall-Associated Receptor Kinase
Source: Plants (Basel). 2023 Oct 17;12(20):3600. doi: 10.3390/plants12203600 (PMC10610323; doi:10.3390/plants12203600)

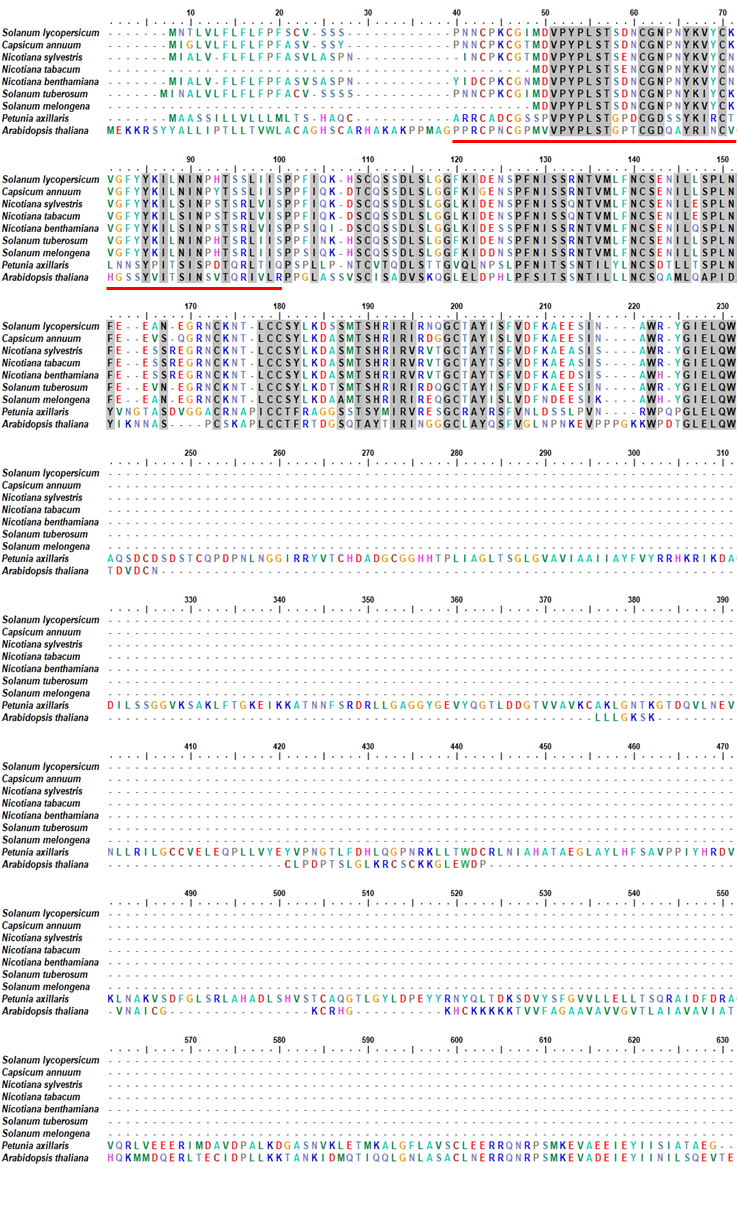

Supplement: Supplementary file 1 [file plants-12-03600-s001.zip › Figure S1.tif]
